# Using a Mobile Health App (ColonClean) to Enhance the Effectiveness of Bowel Preparation: Development and Usability Study

**DOI:** 10.2196/58479

**Published:** 2025-01-08

**Authors:** Hui-Yu Chen, Ming-Hsiang Tu, Miao-Yen Chen

**Affiliations:** 1 Endoscopy Center for Diagnosis and Treatment, Taipei Veterans General Hospital, Taipei City, Taiwan; 2 School of Nursing, National Taipei University of Nursing and Health Sciences, Room B631, No. 365, Ming-te Road, Peitou District, Taipei City, 11219, Taiwan, 886 2 28227101 ext 3186

**Keywords:** mobile health app, bowel preparation, nursing guidance, technology acceptance model, mHealth, mobile health

## Abstract

**Background:**

Colonoscopy is the standard diagnostic method for colorectal cancer. Patients usually receive written and verbal instructions for bowel preparation (BP) before the procedure. Failure to understand the importance of BP can lead to inadequate BP in 25%-30% of patients. The quality of BP impacts the success of colonoscopy in diagnostic yield and adenoma detection. We developed the “ColonClean” mobile health (mHealth) app for Android devices. It incorporates visual representations of dietary guidelines, steps for using bowel cleansing agents, and observations of the last bowel movement. We used the Technology Acceptance Model to investigate whether the use of the ColonClean mHealth app can improve users’ attitudes and behaviors toward BP.

**Objective:**

This study aims to validate the effectiveness of the ColonClean app in enhancing user behavior and improving BP, providing safe and cost-effective outpatient colonoscopy guidance.

**Methods:**

This study uses a structured questionnaire to assess perceived usefulness, perceived ease of use, and users’ attitudes and behaviors toward BP regarding the ColonClean mHealth app. A total of 40 outpatients who were physically and mentally healthy and proficient in Chinese were randomly chosen for this study. The data were analyzed using SPSS 25.0, and we used Pearson product-moment correlation and simple regression analysis to predict the perception of ColonClean.

**Results:**

The results showed that 75% (30/40) of participants achieved an “excellent” or “good” level of BP according to the Aronchick Bowel Preparation Scale. Perceived usefulness and perceived ease of use of the ColonClean mHealth app were positively correlated with users’ attitudes and behaviors (*P*<.05).

**Conclusions:**

The ColonClean mHealth app serves as an educational reference and enhances the effectiveness of BP. Users expressed their willingness to use the app again in the future and recommend it to family and friends, highlighting its effectiveness as an educational guide for BP.

## Introduction

Colorectal cancer (CRC) is the third most prevalent cancer and the second leading cause of cancer-related deaths worldwide [1-3]. Colonoscopy is the standard diagnostic method for colorectal cancer, and adequate bowel preparation (BP) is a necessary and crucial step to effectively examine the entire intestinal mucosa [4,5]. Currently, BP before colonoscopy is primarily provided by nurses through paper-based nursing instructions and oral explanations of relevant precautions [6,7]. This involves dietary restrictions and the use of bowel cleansing agents to remove feces from the colon and facilitate visual examination by physicians [8,9].

The explanations for BP are often lengthy, complex, and difficult to understand and remember. As a result, the proportion of inadequate BP ranges from 25% to 30% [10]. Poor BP could lead to 42% of adenomas and 27% of advanced adenomas not being diagnosed [11,12]. Inadequate BP also leads to a missed detection of 10.55% for sessile polyps [13], which can develop into CRC and are particularly difficult to identify as they lie flat on the mucosal layer of the colon [9]. Incomplete colonoscopy increases the risk of cancer and requires repreparation, adding to health care resource costs [14-16].

The European Society of Gastrointestinal Endoscopy guidelines emphasize that good BP is essential for the diagnosis, treatment, and removal of tumors and precancerous lesions, as well as for the reduction of CRC incidence and its mortality rate. It ensures the quality, safety, and effectiveness of colonoscopy in the clinical examination environment. Adequate BP provides optimal visualization for physicians during the examination, facilitating the detection of polyps or other lesions [10,17].

During the COVID-19 pandemic in 2020, the need for a mobile health (mHealth) app became evident [18,19]. Currently, nursing instructions for BP mainly rely on paper-based materials accompanied by oral explanations, multimedia instruction CDs, or telephone interviews [20]. Using mobile apps can enhance patient education for bowel cleansing [21,22]. Therefore, our research team conducted a literature review and assessed current needs to design and build the “ColonClean” mHealth app for the Android operating system. The purpose of this study is to validate the effectiveness of the ColonClean mHealth app in enhancing users’ attitudes and behaviors toward BP through the Technology Acceptance Model (TAM) by examining the correlations among perceived usefulness, perceived ease of use, usage attitudes, and actual usage behaviors. We aim for the ColonClean mHealth app to serve as a comprehensive and effective outpatient colonoscopy guidance tool, ultimately improving the effectiveness of BP.

## Methods

### Ethical Considerations

This study is an interventional investigation conducted in accordance with research ethics regulations. Approval from the Institutional Review Board of Taipei Veterans General Hospital was obtained prior to the commencement of the study (approval number 2021-10-007AC). The study was conducted from October 27, 2021, to December 31, 2022. Before enrolling participants, the purpose and details of the study were explained to them, and they provided informed consent by signing a consent form. Throughout the research process, all participants’ privacy rights were strictly protected, and the collected data were used solely for research purposes. Participants were not compensated for their participation in this study.

### Study Participants

G*Power 3.1.9.7 for Windows was used to analyze the questionnaire data, considering a power (1-β) of 0.8, α value of .05, a medium effect size of 0.3, and an estimated dropout rate of 20%. As a result, a sample size of 40 participants was determined. A total of 40 outpatients from the gastroenterology and endoscopy center of the medical center were recruited. The inclusion criteria were patients aged 20 years or older, recommended by physicians to undergo colonoscopy, without visual or hearing impairments, and without mental illnesses. After explaining the purpose and content of the study and obtaining written informed consent, paper-based nursing instructions were provided along with verbal explanations of ColonClean, and the BP was conducted. For easy access to ColonClean, the participants downloaded the ColonClean mHealth app on their mobile devices after scanning a QR code. The nursing instruction process involved 1-on-1 teaching, with an explanation lasting approximately 20 minutes in the independent waiting area outside the outpatient visit room. Each research participant was asked to submit feedback on their experience with ColonClean within 3 days of their colonoscopy.

### mHealth App (ColonClean)

Individuals aged ≥50 years are the target group for CRC screening, making them the primary users of the ColonClean mHealth app. Considering the physiological and cognitive decline in older adults, it is crucial to design a user-friendly interface to avoid difficulties in usage. The interface design for older adults incorporates intuitive elements, clear and concise text, and straightforward operational steps [23]. Contrast colors were used for interface color schemes. The operational methods are simple and clear, with functions executed through clicks or straight-line swiping gestures [24,25]. All features were designed to operate within a single page, avoiding menu structures with more than 2 layers of information. This design reduces the psychological pressure on older adults, who may be afraid of making mistakes due to an unfamiliarity with technology; this allows them to focus and read the important information comfortably. Handwritten or voice inputs were preferred for filling in the forms. Clear return or cancel buttons were placed at the top or bottom of the screen. Considering the characteristics of the users, the emphasis was on interaction and feedback rather than simple page browsing to enhance the impression of BP.

The ColonClean mHealth app was developed using the Android 11 operating system due to its low development cost and popularity among the general public [26]. The user interface and content were designed using the Android app package, the Java programming language, and a built-in database.

**Figure 1.**
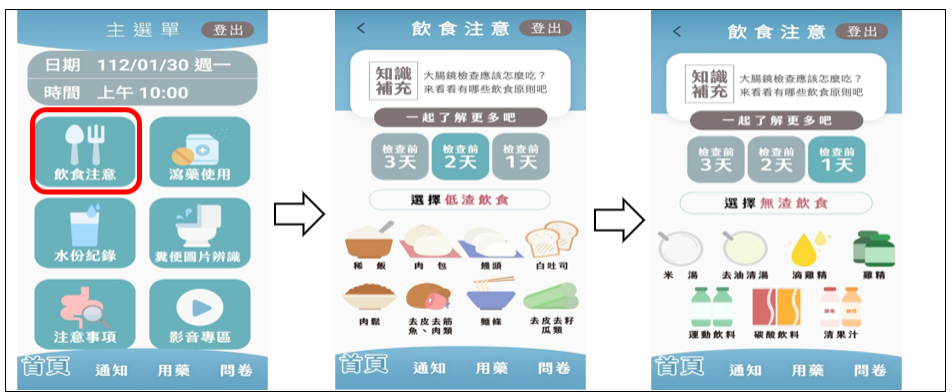
Representative images of “Dietary Options” in ColonClean.

**Figure 2.**
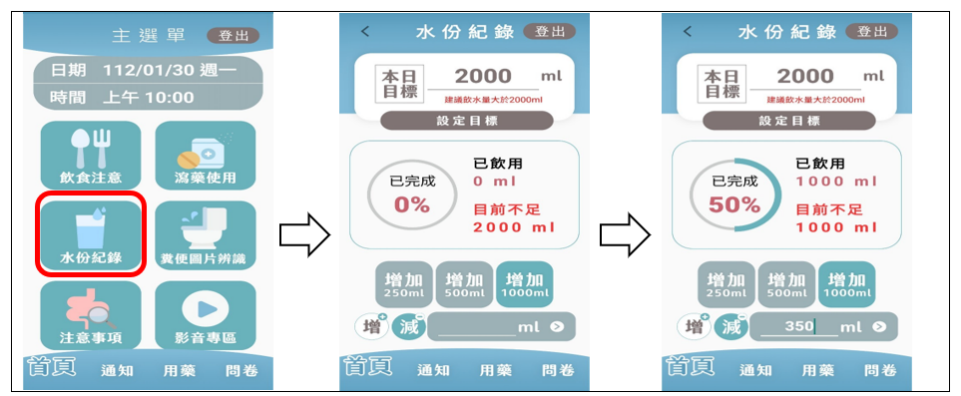
The “Hydration Intake Record” in ColonClean. Bowel preparation requires plenty of fluid intake to facilitate the passage of feces from the bowel.

**Figure 3.**
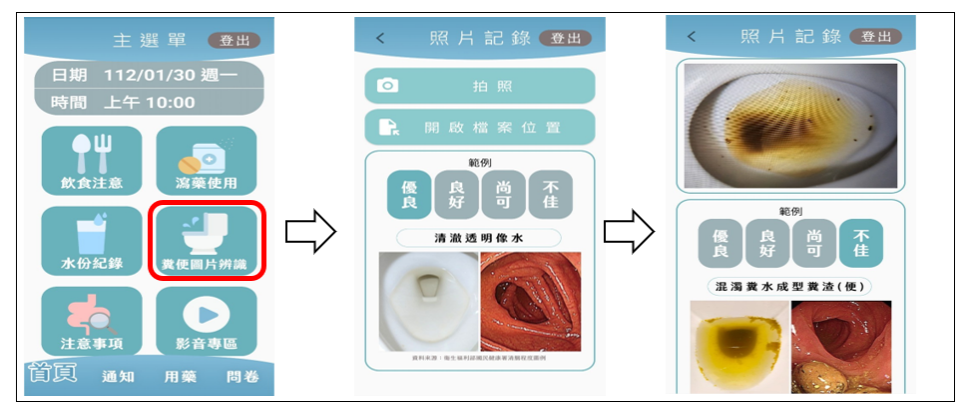
Images of the “Last Bowel Movement Type” section of ColonClean.

The screen display of ColonClean is divided into 3 sections: top, middle, and bottom in a single-page layout for the main screen. The top section displays the “Examination Date and Time.” The middle section features 6 key topics arranged in a zigzag pattern based on their importance. From left to right, these topics include “Dietary Guidelines,” explaining the necessary dietary education starting 3 days before the examination as shown in Figure 1, and “Laxative Use,” explaining the use of bowel cleansing medication. The “Fluid Intake” screen is for recording the amount of fluid consumed (Figure 2), while the “Stool Image Recognition” screen is for providing images to identify the type of the last bowel movement (Figure 3).

“Precautions” explains important points to keep in mind. The bottom section of the screen, from left to right, includes the “Home” button to return to the main screen; “Notifications,” which displays text messages; “Medication,” which provides settings and modifications for regular medication or bowel cleansing agents; and “Questionnaire,” which allows users to provide feedback on their experience using the ColonClean mHealth app.

The ColonClean app sent reminders and notifications to participants in the experimental group starting 3 days before their procedure. These notifications advised participants to begin dietary restrictions, such as avoiding high-fiber fruits and vegetables. Two days before the procedure, the app reminded users to switch to a low-residue diet, focusing on foods like congee or plain white noodles. The day before the procedure, the app prompted users to follow a clear liquid diet, including oil-free broth and sports drinks, and provided instructions on taking BP medications.

The app utilizes tap and swipe gestures for navigation; for user registration, it supports built-in Chinese phonetic input or handwriting recognition on mobile devices. The interface features a fresh color palette, primarily a warm lake green. Key information is highlighted in red, and unnecessary content is removed to offer concise summaries. Unique icons are designed as entry pointers to access relevant content. Tapping on an icon triggers entry into the main menu, which includes dietary guidance, instructions on using bowel cleansing agents (via images and videos), precautions for a colonoscopy, and fluid intake records, and provides references to the type of last bowel movement for understanding the differences compared to that during endoscopy. The design allows users to navigate intuitively, freely scrolling, tapping, and browsing related content between pages [27,28]. Push notifications are recommended to include instructions or prompts. Therefore, through push notifications, users are informed about relevant BP information to avoid any execution errors. Considering the user groups are middle-aged and older adults, complex actions are avoided. Instead, the key content is displayed directly in the push notification message. When a notification is received, users can click on the pushed message, which leads them to the relevant page with clear instructions or recommendations to view more detailed content. This approach reduces the chance of users misunderstanding the message. The user-centered push notification feature for BP steps enhances user engagement [29,30].

### Aronchick Bowel Preparation Scale

After completing a colonoscopy, the physician submits a rating of the BP level using the Aronchick Bowel Preparation Scale (ABPS) in the endoscopy report system [1,31,32]. The ABPS is one of the most comprehensive and commonly used scales to describe the visualization of the colon and assess the percentage of stool covering the entire colon mucosa to explain the state of BP [33]. Based on this assessment, the adequacy of bowel cleansing is graded as excellent, good, fair, poor, or inadequate [11,33-35]. In Taiwan, domestic quality indicators for colonoscopy align with ABPS standards, targeting a 90% or above rating for both “excellent” and “good” categories. This ensures a high standard of care in colorectal screening, enhancing procedural accuracy and improving patient outcomes [36,37].

### TAM

The TAM explains the behavior of users accepting new information technology in the field of computer technology [38]. Perceived usefulness and perceived ease of use affect users’ attitudes toward adopting new technology, thereby influencing willingness and usage behavior. The perceived ease of use, perceived usefulness, attitudes, and behavior of outpatient endoscopy patients toward the ColonClean mHealth app were evaluated to assess their acceptance of using it. The questionnaire adopted a 5-point Likert scale. The 4 dimensions assessed by outpatient endoscopy patients after using ColonClean were perceived ease of use, perceived usefulness, attitudes, and usage behavior. The response options ranged from 1=“strongly disagree,” 2=“disagree,” 3=“neutral,” 4=“agree,” to 5=“strongly agree.” Higher scores indicated a higher level of agreement, while lower scores indicated a lower level of agreement. The questionnaire was initially developed based on literature references [39]. Three nursing informatics experts and scholars were then invited to evaluate the applicability and clarity of each question and to provide suggestions for improvement.

The Cronbach α coefficient was used to assess the internal consistency of the questionnaire items in this study. For the perceived usefulness dimension, consisting of 6 items, Cronbach α was 0.971. For the perceived ease of use dimension, consisting of 6 items, Cronbach α was 0.950. For the attitude dimension, consisting of 5 items, Cronbach α was 0.930. For the usage behavior dimension, consisting of 6 items, Cronbach α was 0.976. The questionnaire comprised a total of 23 items, and the Cronbach α coefficient for the scale was 0.986, indicating high internal consistency, meeting the requirement of a Cronbach α coefficient exceeding 0.7.

The structured questionnaire was designed and validated for reliability and validity by 3 nursing informatics experts. Participants accessed the questionnaire through a built-in link within the app, with the collected data stored in the backend management center for subsequent analysis.

### Analysis

The data were analyzed using SPSS 25.0 (IBM Corp), with the utilization of the Pearson product-moment correlation and simple regression analysis to predict the perception of ColonClean.

## Results

### Demographic Characteristics

The demographic characteristics of the participants were as follows: 17 males (43%) and 23 females (58%); 27 participants aged ≥50 years (68%); and 35 participants with a high school/vocational education or above (88%).

### ColonClean

After completing their colonoscopy, each research participant provided feedback on their experience with the ColonClean app within 3 days.. The feedback highlighted several key aspects of the app’s design that contributed to user satisfaction and engagement:

- User-centered design: Participants appreciated the intuitive user interface, which maintained consistency in themes and main menu content. The clear and simple instructions, presented through a combination of text and visuals, facilitated user-friendly navigation. This consistency helped users feel more confident using the app.
- Examination date reminder: The feature displaying the examination date at the top of the main menu was well-received, as users felt it helped them stay organized and aware of the timing, potentially increasing compliance with preparation protocols.
- Graphic representations: Users found the unique graphic representations of dietary restrictions particularly helpful, as they enhanced understanding compared to traditional text descriptions. This visual approach was seen as effective in clarifying preexamination dietary options.
- Medication instructions: The clear, red-highlighted instructions for using bowel cleansing agents, along with drug images, dosage information, and timing, received positive feedback. Users appreciated the visual cues that simplified the medication process.
- Hydration tracking: Participants valued the hydration tracking feature, which encouraged them to monitor their water intake. Reminders to increase consumption when below recommended levels and positive feedback for meeting goals were highlighted as motivational elements that enhanced their commitment to the BP process.
- Bowel movement comparison: The option to take a photo of their last bowel movement and compare it with reference images was particularly beneficial. Users reported that this feature provided clarity on the required level of bowel cleanliness, enhancing their understanding of preparation requirements.
- Medication reminders and push notifications: The integration of medication reminders, particularly for chronic medications and anticoagulant discontinuation, was praised for preventing potential issues during the colonoscopy process. Users appreciated the timely push notifications that kept them informed about important tasks related to their preparation.

Overall, the research results indicated that users found the design of the ColonClean app effective in enhancing their understanding of and compliance with BP instructions. The app’s focus on intuitive navigation, visual aids, and timely reminders contributed to a positive user experience, ultimately improving the effectiveness of BP for colonoscopy.

### ABPS

After completing the colonoscopy, the BP level is reported by the endoscopist using the ABPS: 10 individuals (25%) achieved a “fair” level, 26 individuals (65%) achieved a “good” level, and 4 individuals (10%) achieved an “excellent” level. There were no cases of “poor” or insufficient preparation leading to interruption or inability to perform the examination.

### TAM

#### Perceived Usefulness

The details of perceived usefulness are shown in Table 1.

**Table 1.** Perceived usefulness of the ColonClean app.

Perceived usefulness of the ColonClean app	Mean (SD)
Using ColonClean helps me quickly understand the tasks required for bowel preparation.	4.60 (0.59)
Using ColonClean provides easy access to relevant information about bowel preparation.	4.55 (0.68)
I believe that using ColonClean can improve the accuracy of my bowel preparation.	4.65 (0.53)
Using ColonClean enables me to effectively complete the required bowel preparation tasks.	4.65 (0.48)
ColonClean provides clear reminders about bowel preparation tasks, such as dietary restrictions and monitoring the type of my last bowel movement.	4.60 (0.59)
I find the educational information provided by ColonClean sufficient for completing my bowel preparation tasks.	4.65 (0.53)

The table presents users’ perceptions of the usefulness of the ColonClean app in managing BP tasks. The mean scores, averaging around 4.62 of 5, suggest that users generally find the app very helpful. Below is an interpretation of the key findings:

- Understanding tasks (mean 4.60, SD 0.59): Users feel that the ColonClean app helps them quickly understand the requirements for BP, indicating clear and effective communication of instructions.
- Access to relevant information (mean 4.55, SD 0.68): The app is seen as a convenient tool for accessing important information about the preparation process. However, this metric has a slightly lower score, suggesting minor room for improvement in delivering information.
- Increased accuracy (mean 4.65, SD 0.53): Users believe that the app enhances the accuracy of their BP, reflecting a high level of trust in the app’s guidance to meet medical standards.
- Effective completion of tasks (mean 4.65, SD 0.48): The app is viewed as highly effective in helping users complete BP tasks correctly, suggesting that it provides practical support throughout the process.
- Clear reminders (mean 4.60, SD 0.59): The app is viewed as highly effective in helping users complete BP tasks correctly, suggesting that it provides practical support throughout the process.
- Sufficient educational information (mean 4.65, SD 0.53): The app provides enough educational material for users to feel confident about completing their preparation, further supporting its educational role.

In summary, the ColonClean app was perceived as highly useful across various aspects of the BP process, with particularly strong scores for accuracy, task completion, and educational sufficiency. These results highlight the app’s effectiveness in improving the user experience and promoting adherence to BP protocols.

#### Perceived Ease of Use

The details of perceived ease of use are shown in Table 2.

**Table 2.** Perceived ease of use of the ColonClean app.

Perceived ease of use of the ColonClean app	Mean (SD)
I think the interface design of ColonClean is clear and easy to understand.	4.63 (0.54)
I find the process of using ColonClean to be smooth.	4.63 (0.54)
It is easy to navigate and locate the desired functions in ColonClean.	4.63 (0.54)
I think the data presentation in ColonClean is quick and stable.	4.65 (0.58)
I find the font size in the ColonClean interface appropriate and easy to read.	4.53 (0.75)
The information provided by ColonClean makes it easier to understand the bowel preparation tasks.	4.68 (0.53)

Table 2 presents users’ perceptions of the ease of use of the ColonClean app, highlighting their experiences with various interface features. The mean scores indicate a generally high level of satisfaction, with most ratings above 4.62 of 5, suggesting that users find the app intuitive and user-friendly. Below is an interpretation of the key findings:

- Clear interface (mean 4.63, SD 0.54): Users feel that the interface design is intuitive and user-friendly. This clarity in design is crucial for users’ initial engagement with the app, suggesting that the developers prioritized usability.
- Smooth user experience (mean 4.63, SD 0.54): The app operates smoothly, without lag or issues, which is essential for encouraging continued use and satisfaction with digital health tools.
- Easy navigation (mean 4.63, SD 0.54): The ease of navigation suggests that users can quickly find the features they need, reducing frustration and enhancing overall satisfaction.
- Quick and stable data presentation (mean 4.65, SD 0.58): Users appreciate the app’s rapid and reliable presentation of information. This reliability enhances user trust, which is particularly important in health care applications where accuracy is critical.
- Appropriate font size (mean 4.53, SD 0.75): Although the font size received a slightly lower rating, it still indicates that most users find it suitable. The slightly lower score suggests that some users might prefer larger text or different font styles, which could be an area for improvement.
- Enhanced understanding of BP (mean 4.68, SD 0.53): Users find that the information provided by ColonClean significantly aids their understanding of BP tasks, which is essential for ensuring compliance with medical procedures.

In summary, the high average scores across these metrics indicate that users generally find ColonClean to be an easy-to-use app that effectively supports BP. The highlighted areas demonstrate strengths in design, functionality, and educational support, with only minor adjustments needed in font size to further enhance the user experience. These findings suggest that ColonClean is a valuable tool for improving patient preparation and potentially contributing to better clinical outcomes.

#### Attitudes Toward Usage

The details of attitudes toward usage are shown in Table 3.

**Table 3.** Attitudes toward usage of the ColonClean app.

Attitudes toward usage of the ColonClean app	Mean (SD)
I believe ColonClean is an excellent choice for assisting with bowel preparation before an examination.	4.63 (0.54)
I am currently satisfied with the benefits provided by using ColonClean.	4.65 (0.53)
I would be willing to use ColonClean again in the future if I need to undergo bowel preparation.	4.63 (0.59)
I find the information provided by ColonClean to be very useful.	4.60 (0.59)
I prefer using ColonClean over paper-based nursing instructions (single sheets).	4.35 (0.77)

Table 3 summarizes users’ attitudes toward the use of the ColonClean app, reflecting generally positive perceptions of its utility and effectiveness in assisting with BP tasks. The mean scores indicated a high level of satisfaction, with most ratings above 4.57 of 5, suggesting a strong endorsement of the app’s features and benefits. Below is an interpretation of the key findings:

- Good choice for assistance (mean 4.63, SD 0.54): This high mean score reflects a strong consensus among users that ColonClean is an effective tool for supporting BP. The low SD suggests consistent agreement on its usefulness in this context.
- Satisfaction with benefits (mean 4.65, SD 0.53): Users’ satisfaction indicates that the app meets their expectations and provides meaningful benefits during BP. The relatively low SD further shows that most users feel positively about the app’s contributions.
- Willingness to use again (mean 4.63, SD 0.59): Users expressed a strong intention to use ColonClean for future BPs, suggesting that their initial positive experiences built trust in the app and that they see it as reliable.
- Usefulness of information (mean 4.60, SD 0.59): The perception that ColonClean provides highly useful information reinforces its role as a valuable educational resource, effectively aiding users in understanding the preparation process.
- Preference over paper instructions (mean 4.35, SD 0.77): Although users generally prefer ColonClean over traditional paper-based instructions, the slightly lower score and higher SD suggest that some users may still value printed materials or have mixed feelings about fully transitioning to digital formats. This variability presents an opportunity for further enhancements in the app’s usability and content delivery.

In summary, the high mean scores (above 4.57) suggested that users had a positive attitude toward ColonClean, seeing it as an effective and beneficial tool for BP. The consistent responses (low SDs) indicated broad agreement among users on its usefulness. Meanwhile, the variation in preference over paper instructions may highlight areas for potential improvement. This feedback can guide future updates and enhancements to maximize user satisfaction and efficacy.

#### Usage Behavior

The details of usage behavior are shown in Table 4.

**Table 4.** Usage behavior of the ColonClean app.

Usage behavior of the ColonClean app	Mean (SD)
I am willing to use ColonClean as an aid for bowel preparation.	4.73 (0.45)
I am highly satisfied with the ability to access knowledge through ColonClean during bowel preparation.	4.70 (0.52)
I would recommend ColonClean to friends and family in the future.	4.63 (0.63)
I would use ColonClean to improve the effectiveness of bowel preparation.	4.70 (0.52)
I believe ColonClean provides useful information during the bowel preparation period.	4.68 (0.53)
Overall, I find using ColonClean to be satisfactory.	4.73 (0.51)

Table 4 summarizes the usage behavior of the ColonClean app and reveals overwhelmingly positive user sentiment. The overall mean score of 4.69 indicates high satisfaction with the experience of using ColonClean. This average score, combined with a relatively low SD (0.50), suggests a strong consensus among users regarding the app’s effectiveness and utility. Below is an analysis of the meaning behind the scores:

- Willingness to use (mean 4.73, SD 0.45): The high mean score indicates that users are very inclined to use ColonClean as a supportive tool for BP. The low SD signifies that this sentiment is widely shared, suggesting strong confidence in the app’s benefits.
- Satisfaction with knowledge access (mean 4.70, SD 0.52): Users expressed a high level of satisfaction with the knowledge accessible through the app during BP. This indicates that ColonClean effectively meets their informational needs, enhancing their preparation experience.
- Recommendation to others (mean 4.63, SD 0.63): The willingness to recommend ColonClean to friends and family reflects users’ trust in the app’s efficacy. Although this score remained positive, the slightly higher SD suggests some variability in users’ willingness to recommend it, possibly due to differing experiences or expectations.
- Improvement of effectiveness (mean 4.70, SD 0.52): Users believed that ColonClean enhanced the effectiveness of their BP, reinforcing the app’s perceived value as a helpful resource in the preparation process.
- Useful information obtained (mean 4.68, SD 0.53): The belief that the app provides useful information further underscores its utility as an educational tool. Users recognized that the app offers relevant guidance throughout the preparation period.
- Overall satisfaction (mean 4.73, SD 0.51): The high overall satisfaction score indicates that users felt positively about their experience with ColonClean. The consistency of this sentiment, reflected by the low SD, suggests that many users share a similar level of satisfaction.

In summary, the high mean scores across all categories reflect users’ positive attitudes and satisfaction, underscoring ColonClean as a valuable tool for assisting with BP. The low SDs across most items indicate consistency in user experiences, suggesting that ColonClean effectively meets users’ needs and expectations. This feedback is critical for developers, as it reinforces the app’s strengths and highlights areas where further enhancements could be beneficial.

### Analysis

#### Pearson Correlation Coefficients

Pearson correlation coefficients were used to analyze the relationships among users’ attitudes, perceived usefulness, perceived ease of use, and usage behavior of the ColonClean mHealth app (Table 5). The results indicated a significant positive correlation between users’ attitudes and perceived usefulness (*r*_38_=.907, *P*<.001), users’ attitudes and perceived ease of use (*r*_38_=.825, *P*<.001), users’ attitudes and usage behavior (*r*_38_=.835, *P*<.001), perceived usefulness and perceived ease of use (*r*_38_=.894, *P*<.001), perceived usefulness and usage behavior (*r*_38_=.933, *P*<.001), and perceived ease of use and usage behavior (*r*_38_=.958, *P*<.001). Positive correlations were demonstrated between perceived usefulness and perceived ease of use, usage attitude, and usage behavior.

**Table 5.** Correlations among perceived usefulness, perceived ease of use, usage attitude, and usage behavior.

		Perceived usefulness	Perceived ease of use	Usage attitude	Usage behavior
**Perceived usefulness**					
	Pearson correlation analysis	1			
	Significance level (2-tailed)				
	Number	40			
**Perceived ease of use**					
	Pearson correlation analysis	0.894	1		
	Significance level (2-tailed)	<.001			
	Number	40	40		
**Usage attitude**					
	Pearson correlation analysis	0.907	0.825	1	
	Significance level (2-tailed)	<.001	<.001		
	Number	40	40	40	
**Usage behavior**					
	Pearson correlation analysis	0.933	0.958	0.835	1
	Significance level (2-tailed)	<.001	<.001	<.001	
	Number	40	40	40	40

#### Simple Regression Analysis

We used simple regression analysis to predict the correlation among the perceived usefulness, perceived ease of use, usage attitude, and usage behavior in the context of ColonClean. The correlation between perceived usefulness and usage attitude, with usage attitude as the criterion variable and perceived usefulness as the predictor variable, yielded B=0.907, *t*_38_=13.3. This model explained 82.3% of the variance in usage attitude (*R*^2^=0.823), and the test result with *P*<.001 indicated that perceived usefulness was a significant predictor variable for usage attitude. This suggests that when users perceive the app as providing substantial assistance, their attitude toward using it improves significantly. This highlights the importance of enhancing the app’s practical functionality to foster a positive user experience. Similarly, the correlation between perceived usefulness and usage behavior, with usage behavior as the criterion variable and perceived usefulness as the predictor variable, resulted in B=0.933, *t*_38_=16.0. This model explained 87.1% of the usage behavior (*R*^2^=0.871), and the test result with *P*<.001 demonstrated that perceived usefulness significantly predicted usage behavior. When users believe that the app helps improve their preparation process, they are more likely to engage actively with it. The correlation between perceived ease of use and usage attitude, with usage attitude as the criterion variable and perceived ease of use as the predictor variable, resulted in B=0.825, *t*_38_=9.0. This explained 68% of the variance in attitude toward use (*R*^2^=0.68), with *P*<.001, indicating perceived ease of use as a significant predictor of attitude toward use. When users find the app easy to use, their attitude becomes more positive. This reflects their comfort and satisfaction during use, which in turn influences their willingness to engage with the app. When determining the correlation between perceived ease of use and usage behavior, with usage behavior as the criterion variable and perceived ease of use as the predictor variable, the result was B=.958, *t*_38_=20.5. This model explained 91.7% of the variance in usage behavior (*R*^2^=.917), with *P*<.001, indicating that perceived ease of use significantly predicted usage behavior. When users perceive the app as easy to operate, they are more likely to engage actively. This underscores the importance of simplifying the app’s interface and functionality to enhance user engagement and satisfaction.

In summary, the results indicate that both perceived usefulness and perceived ease of use have a significant positive impact on user attitudes and behaviors. This suggests that enhancing the perceived usefulness and ease of use of the ColonClean app during its design may encourage more positive user attitudes and behaviors, thereby improving health management outcomes. These findings not only validate the app’s usability but also provide empirical support for future improvements.

## Discussion

### Principal Findings

Mobile health apps are increasingly used for illness prevention, education, and promoting healthy lifestyles [40]. However, the requirements for BP are often complex and challenging for patients to navigate and remember. To help outpatient colonoscopy patients adhere to dietary guidelines, manage bowel cleansing agents, and maintain regular medication schedules, push notifications were incorporated into the app’s design. The app, protected under the patent titled “ColonClean Mobile Management System” (patent number M639094, Republic of China), features a user-centric interface that is both intuitive and visually appealing. The well-designed interface, content, push notifications, and multimedia section of the ColonClean mHealth app provide users with a convenient and helpful experience when using their mobile devices for health management. Users expressed their willingness to use the app again in the future and recommend it to family and friends, highlighting its effectiveness as an educational guide for BP.

### User Attitudes and Preference for Paper-Based Instructions

Despite the overall positive reception of the ColonClean app, some users indicated a preference for paper-based instructions, as reflected in the lower mean score (mean 4.350.77) on this aspect. This finding aligns with existing literature suggesting that certain users may find physical formats more reliable or easier to reference than digital alternatives [41]. Factors contributing to this preference may include concerns about technology accessibility, device compatibility, or personal comfort with digital apps for health management.

Additionally, the preference for paper-based instructions may arise from a desire for tangible resources that can be easily annotated or highlighted [42]. Some users may feel that printed materials provide a more straightforward and immediate reference during the preparation process, particularly in situations where mobile devices are not accessible or convenient [43]. Therefore, while the digital format of the ColonClean app offers numerous advantages, such as interactivity and real-time updates, it is essential to recognize the value that traditional paper-based resources continue to hold for certain individuals.

### Limitations and Recommendations

The ColonClean app operates on the Android system and functions as an mHealth app for mobile devices. However, it is important to note that during the acceptance process, instances may arise where individuals with non-Android operating systems or outdated device models are unable to be included in this study. Those unable to download ColonClean by scanning the QR code were excluded. It is advisable to consider optimizing future mHealth apps so that they can be integrated into mobile devices of various operating systems. This enhancement would contribute to more inclusive nursing guidance for BP, ensuring accessibility across platforms.

In the current era of smart health care, it is recommended to incorporate artificial intelligence recognition capabilities into the ColonClean app. Specifically, for the section where users capture images of their last bowel movement, artificial intelligence can automatically analyze and determine the quality of the stool, categorizing it as “excellent,” “good,” “fair,” or “poor.” This improvement would facilitate a more accurate assessment of the final bowel movement status. Furthermore, optimizing the ColonClean mHealth app for other individuals who require BP, such as those undergoing colorectal surgery or hospitalized patients preparing for colonoscopy, would ensure the successful completion of bowel cleansing.

Additionally, in light of the COVID-19 pandemic, when individuals undergoing screening needed to reschedule their appointments due to a positive diagnosis or being a close contact, or when they encountered issues like poor bowel movements, a consultation chat window was recommended as a future addition to address user inquiries and provide real-time solutions, thus maintaining user engagement and satisfaction. To ensure the continuity of examinations and achieve the objectives of examination, diagnosis, and treatment, it is crucial to minimize ineffective examinations or schedule cancellations that may impact individuals in need of these examinations. This approach aims to maintain a steady number of examinations and fulfill the purpose of providing necessary medical assessments and subsequent care.

### Conclusions

In summary, while the ColonClean app shows promise in supporting BP, it is essential to recognize user attitudes toward traditional paper-based instructions. Enhancing the app with broader accessibility, artificial intelligence capabilities, and additional user support features could significantly improve its effectiveness and reach, ultimately contributing to better health outcomes for patients requiring BP.

## Abbreviations

ABPS: Aronchick Bowel Preparation Scale
BP: bowel preparation
CRC: colorectal cancer
mHealth: mobile health
TAM: Technology Acceptance Model
